# From anemia to polycythemia in 4 weeks

**DOI:** 10.1002/ccr3.879

**Published:** 2017-08-03

**Authors:** Omer A. Hassan, Melissa Y. Y. Moey, Christos N. Papageorgiou

**Affiliations:** ^1^ Saba University School of Medicine The Bottom Saba Dutch Caribbean The Netherlands; ^2^ SSM Cancer Care St. Mary's Health Center St. Louis Missouri USA; ^3^ Division of Hematology and Oncology Department of Internal Medicine Saint Louis University School of Medicine St. Louis Missouri USA; ^4^ Present address: Department of Pathology Wake Forest School of Medicine Medical Center Boulevard Winston‐Salem North Carolina 27157 USA; ^5^ Present address: Department of Internal Medicine Vidant Medical Center/East Carolina University 600 Moye Blvd Greenville North Carolina 27834 USA

**Keywords:** Anemia, autoimmune disease, polycythemia vera, rheumatoid arthritis, thrombotic thrombocytopenic purpura

## Abstract

Primary polycythemia (PCV) may coexist in otherwise asymptomatic patients particularly in the presence of unsuspecting conditions such as Thrombotic thrombocytopenic purpura (TTP). In presumed “idiopathic TTP,” autoimmune conditions such as rheumatoid arthritis (RA) should be investigated as a possible etiology for TTP. Standardization of targeted therapy with immunomodulatory agents may be recommended for this subset of patients.

## Introduction

Thrombotic thrombocytopenic purpura (TTP) is a consumptive coagulopathy that is typically associated with the laboratory findings of microangiopathic hemolytic anemia, thrombocytopenia, and renal insufficiency resulting in generalized symptoms of fever, fatigue, mental status changes, headache, and easy bruisability 1. The more common secondary form of TTP occurs after infection, drugs, toxins, and occasionally autoimmune diseases as seen in reported cases of systemic lupus erythematosus (SLE), Sjögren's, and mixed connective tissue disease 2, 3, 4, 5. TTP in association with rheumatoid arthritis (RA) is extremely rare as there has only been one reported case published to date 6.

Primary polycythemia (PCV) is a BCR‐ABL1‐negative myeloproliferative neoplasm involving an increase in cellular proliferation of the myeloid lineage, particularly erythroid precursors, that often affects males more than females at a median age of 60 years old 1, 7. In PCV, greater than 90% of patients have the acquired janus kinase 2 (JAK2) V617F mutation 8, 9, causing activation of the intrinsic JAK/STAT (signal transducers and activators of transcription) pathway 8, 9. The resultant hyperviscosity in polycythemia leads to the reported symptoms typically seen in PCV such as fatigue, pruritus, night sweats, bone pains, fever, and weight loss 10. PCV and TTP have been classically observed as two separate entities rarely occurring together and each with its own unique pathophysiology. We report the first case of newly diagnosed PCV within 4 weeks of a relapsing TTP episode with concurrent undiagnosed RA. As both hematologic diseases pose risks for thrombosis, we discuss the challenges of managing thrombotic complications in such patients.

## Case Presentation

A 63‐year‐old African American male with a past medical history of former tobacco use, sickle cell trait, hypertension, transient ischemic attack (TIA), chronic joint pain, and prostate cancer treated with radiation therapy was admitted because of a 3‐week history of progressively worsening weakness, confusion, intractable lower extremity stiffness, fever, and easy bruising. He presented with severe anemia with a hematocrit (Hct) of 25.3%, severe thrombocytopenia with a platelet count of 12 × 10^9^/L, hyperbilirubinemia with total bilirubin of 1.7 mg/dL, creatinine of 1.7 mg/dL, lactate dehydrogenase >900 IU/L, and undetectable serum haptoglobin. A peripheral blood smear was obtained that confirmed marked thrombocytopenia and showed scattered nucleated red cells, polychromasia, and rare blasts without evidence of acute leukemia. He reported a history of at least three previous episodes of thrombocytopenia accompanied by flulike symptoms, lightheadedness, and hematuria, which at least one was treated successfully at an outside facility with sole therapy of immunosuppressive agents. From his history of prior recurrent thrombocytopenia with corresponding remission on high‐dose steroid therapy alone, it was unclear whether he initially had TTP or possibly another process such as immune thrombocytopenic purpura (ITP). The possibility of malignancy‐related microangiopathy was unlikely at this point given the fact that his prostate cancer was in remission.

A direct antiglobulin test to rule out autoimmune hemolytic anemia was negative. As the patient fit the classic pentad for TTP, ADAMTS13 (a disintegrin and metalloproteinase with a thrombospondin type 1 motif, member 13) activity was measured and subsequently determined to be <5% which confirmed the diagnosis of TTP. In light of his chronic joint pain and worsening stiffness, acquired TTP from concomitant rheumatologic/autoimmune disease was suspected. He was started immediately on daily one‐volume plasma exchange (PEX) and steroid therapy with an excellent response during outpatient visits as normalization of Hct, platelet count, LDH, ADAMTS13 activity was achieved with a total of nine sessions. The patient was subsequently referred to a rheumatologist where he was subsequently diagnosed with seropositive RA based on a positive rheumatoid factor, anticyclic citrullinated peptide antibody and antinuclear antibody blood screen with a titer of <1:40 and a speckled pattern.

Two weeks after discharge, he presented for follow‐up at the clinic where he was fully alert and oriented, energetic without any physical or hematological abnormalities and a peculiar finding on complete blood count (CBC) of erythrocytosis. His Hct, repeated twice, was confirmed to be raised at 55.8%. On a subsequent follow‐up 2 weeks later, while on a prednisone taper, the patient's Hct continued to climb and reached a high of 56.5% with CBC only notable for steroid‐induced leukocytosis. The patient had no complaints of chest pain, night sweats, pruritus, headache, dizziness, vision changes or numbness, tingling, burning or weakness in hands, feet, arms, and legs. Ultrasound imaging of the abdomen revealed a normal size spleen without any renal abnormally. The patient's wife however brought to attention the patient's heavy snoring during the evening that was associated with apneic episodes. At this point, taking into consideration the patient's smoking history and the absence of splenomegaly or thrombocytosis, erythropoietin (EPO) level was measured, which was surprisingly found to be 6.8 mIU/mL. Whole blood viscosity was elevated at 7.4 cP.

**Figure 1:**
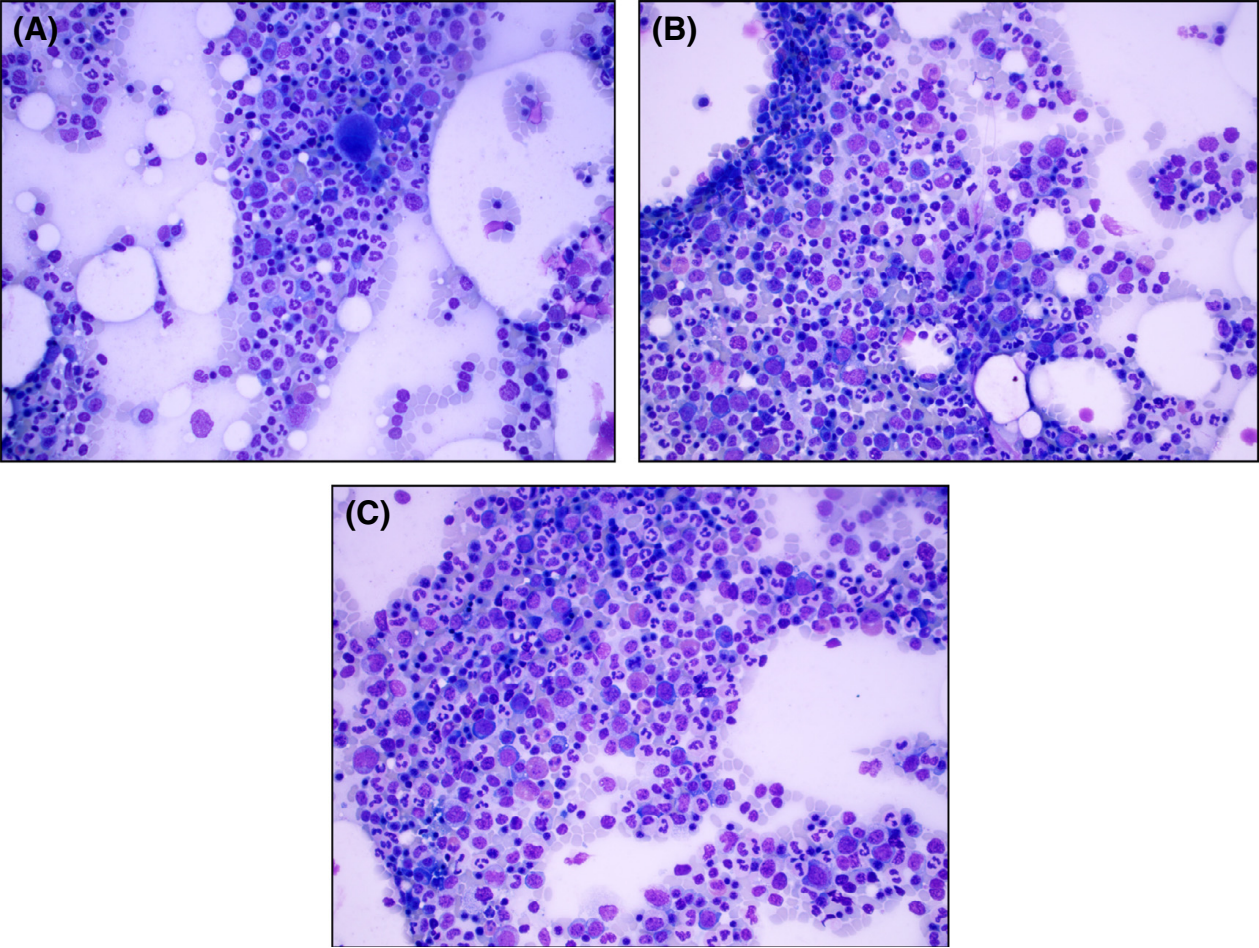
Bone marrow biopsy. (A–C) The bone marrow aspirate smears are cellular with significant erythroid hyperplasia (57% are erythroid precursors on differential) and also erythroid dyspoiesis. The myeloid cells show no significant dyspoiesis. Blasts are not increased.

As the EPO level was within low‐normal range, both primary polycythemia and secondary polycythemia (particularly obstructive sleep apnea, OSA) were considered in the differential diagnoses. Polysomnography studies showed evidence of nocturnal hypoxemia and severe OSA based on apnea–hypopnea index of 99.7 events per hour of sleep. A bone marrow (BM) biopsy and polymerase chain reaction (PCR) detection JAK2 V16F mutation were additionally performed to exclude PCV. A BM biopsy revealed normocellular marrow of 40% with erythroid hyperplasia, decreased iron storage noted as 1 of 4+, mild dyserythropoiesis, and no evidence of lymphoma or leukemia (Fig. 1). In a peripheral blood screen, the JAK2 V617F mutation was detected by real‐time PCR amplification of genomic DNA. Cytogenetics showed no evidence of clonal abnormalities, and fluorescence in situ hybridization (FISH) was negative for myeloproliferative disease probes.

Owing to the rather abrupt diagnosis of PCV in concurrence with a history of frequently relapsing TTP with severe ADAMTS13 deficiency (<5% activity), prior episode of TIA and age greater than 60 years old, the patient was immediately started on a daily low‐dose aspirin (ASA) for thromboprophylaxis. Weekly phlebotomy sessions were planned in conjunction with daily 500 mg hydroxyurea cytoreductive therapy in addition to starting continuous positive airway pressure (CPAP) for his OSA.

The patient was followed up weekly thereafter with routine laboratory studies showing normalization of his Hct to 46% after 4 weeks of initial diagnosis of PCV, including normal platelet and WBC counts. He received a total of four phlebotomy sessions, and hydroxyurea was reduced to 500 mg every other day after 4 weeks due to treatment‐related transaminasemia and intolerability while continuing on low‐dose ASA. With respect to the patient's TTP, he experienced one episode of isolated mild thrombocytopenia 12 weeks after the initial diagnosis which was presumed to be an early relapse and was successfully treated with prompt PEX and high‐dose steroids in addition to a total of 4‐week course of rituximab 375 mg/m^2^ once per week. The response to therapy was indicated by repeat CBCs showing normalization of platelet count. ADAMTS13 level was measured again approximately 2 weeks after the last pheresis, which was within normal limits. After approximately 6 months of follow‐up with biweekly surveillance, the patient's PCV and TTP was monitored with monthly CBC, CMP, haptoglobin, and bimonthly ADAMTS13 levels. In the event of a decrease in ADAMTS13 activity <20%, then preemptive administration of a 4‐week course of rituximab was considered per TTP protocol. The patient is in remission to date and continues to follow up at the clinic.

## Discussion

The diagnosis of PCV within 4 weeks of a life‐threatening TTP relapse in the setting of undiagnosed RA was unique to our patient and to our knowledge is the first reported case of such a peculiar association. It was a particularly surprising finding as he was asymptomatic and devoid of physical findings despite his elevated Hct. Given our patient's benign physical examination together with his smoking history lead to further investigation with measurement of EPO levels for the possibility of any secondary causes of polycythemia. EPO was surprisingly in the low‐normal range of 6.4 mIU/mL. Subnormal ranges of EPO are seen in about 81% of patients with PCV with a general understanding of the unreliability of EPO levels alone in differentiating between primary versus secondary polycythemia 11.

Therefore, in order to diagnose PCV, specific criteria must be met which include a Hct >52% in men and JAK2V617F or other functionally similar mutations such as JAK2 exon 12 or 14 mutation in addition to either a BM biopsy showing hypercellularity for age with trilineage growth of prominent erythroid, granulocytic, and megakaryocytic proliferation or serum EPO levels below the reference range for normal 12. The finding of the acquired JAK2 V617F mutation and erythroid hyperplasia on BM biopsy allowed us to diagnose PCV in our asymptomatic patient despite a normal EPO level.

The occurrence of PCV with concomitant OSA created an additional challenge of differentiating between the inciting factors for his elevated Hct. It is possible that his OSA may have been a contributing factor in normalizing his EPO level, essentially “masking” his underlying PCV. This is different from reports of masked PCV (mPV) as described by Barbui and colleagues where patients were found to be predominantly men with lower Hgb cutoff of 16.5 mg/dL, higher platelet counts, and BM biopsy showing reticulin fibrosis 13, 14.

Another unique observation in our patient was the presence of undiagnosed RA amidst his relapsing TTP episodes. The role of autoimmunity as secondary causes in the pathogenesis of TTP has recently received more attention over the past few years with studies documenting a high occurrence of SLE and other autoimmune disorders with TTP 4, 5. Literature review to date reveals only one case report of an isolated TTP event in association with RA 6. This is supported by the observation of ADAMTS13 inhibitor (immunoglobulin IgG, IgG4 subclass) in a large proportion of cases of idiopathic and secondary TTP.

We suspect that the proportion of patients with an underlying autoimmune condition inciting TTP may be underrepresented. This could be due to the apparent lack of symptoms suggestive of any autoimmune disease to necessitate further investigation for autoimmunity at the time of TTP diagnosis. There were several indicators of a possible autoimmune source for our patient's TTP which was his complaints of chronic joint pains and worsening stiffness in addition to the observed increase in ADAMTS13 activity to normal range following PEX and steroid therapy. It is therefore important to maintain a high degree of suspicion of underlying autoimmune disorder in patients with “idiopathic” TTP. We recommend implementing a multidisciplinary approach involving both hematology and rheumatology in investigating unidentifiable causes of TTP in order to stratify patients based on specific etiological parameters such as autoimmunity.

Our patient's rather abrupt diagnosis of PCV after an episode of relapsing TTP raised an unprecedented challenge in the management of both short‐ and long‐term thrombotic complications. The pathogenesis of TTP is multifactorial, proposed to involve endothelial cell injury from release of inflammatory cytokines such as TNF‐α, IL‐6, and CRP that initiate the release of unusually large multimers of von Willebrand factor (ULvWF) and cause perturbations in the ADAMTS13 molecule leading to autoimmunization and subsequent failure of ULvWF disintegration 15, 16. It is in fact the presence of all of these fundamental components involved in the pathogenesis of TTP with a “double hit” from his co‐existing PCV and RA: (1) hyperviscosity from PCV causing endothelial cell injury; (2) upregulation of cellular factors in leukocytes and platelets favoring thrombosis as with the JAK2 V617F mutation; and (3) proinflammatory cytokine release and autoimmunization as part of the systemic inflammatory response in RA that made this case particularly challenging. This sets our patient at an even higher risk of thrombotic events due to the synergistic effects of his PCV and underlying RA, than individuals solely with a history of TTP and consequently eludes to the debate of more aggressive treatment options in the prevention of thrombosis.

In terms of maintenance therapy for TTP, there is no known effective therapy for the prevention of relapses. While stronger immunosuppressive agents such as rituximab, cyclophosphamide, vincristine, and azathioprine have been administered in cases of refractory and relapsing TTP with some degree of success 16, there is still a growing need for larger clinical trials to appropriately assess their efficacy and safety in this unique subgroup of patients. Taking this into consideration, we decided to implement standard of care measurements agreeable to literature with PEX plus high‐dose steroids, and considered rituximab for preemptive treatment based on repeat ADAMTS13 activity level or in the case of future relapse. Prophylactic treatment of thrombosis with low‐dose ASA, phlebotomy, and hydroxyurea was recommended based on current guidelines for a very high‐risk patient, whose age is greater than 60 years and with a prior history of thrombotic events 1.

## Conclusion

In conclusion, this rare case of concurrent PCV and TTP occurring in the midst of undiagnosed RA exemplifies the challenges in preventing thrombotic complications. Our clinical thought process throughout this complex case reflects the growing need for a more comprehensive risk assessment of thrombosis in patients with PCV, even more so in patients with concomitant conditions that predispose to thrombosis including autoimmune disorders.

## Authorship

OAH: performed literature review and preparation of the manuscript. MYYM: contributed to the preparation of the manuscript. CNP: supervised project and reviewed manuscript.

## Conflict of Interest

None declared.
